# Association of perioperative step count tracked by a wristband with surgical outcomes in minimally invasive lung cancer surgery: a prospective observational study

**DOI:** 10.3389/fmed.2025.1590327

**Published:** 2025-07-29

**Authors:** Yuanyuan Yao, Ying Wang, Yi Liu, Yang Yu, Xuena Wang, Tingting Wang, Bin Zheng, Min Yan

**Affiliations:** ^1^Department of Anesthesiology, The Second Affiliated Hospital, Zhejiang University School of Medicine, Hangzhou, China; ^2^School of Anesthesiology, Weifang Medical University, Weifang, China; ^3^Department of Anesthesiology, The First People’s Hospital of Huzhou, Huzhou, China; ^4^Department of Surgery, University of Alberta, Edmonton, AB, Canada

**Keywords:** lung surgery, patient recovery, physical activity, step count, surgical outcome, wearable device

## Abstract

**Background:**

Physical activity has been reported to be associated with surgical outcomes, but most previous studies have focused solely on postoperative step counts. To better understand the relationship between step count at different phases and surgical outcomes, we prospectively recorded patients’ step counts before and after lung surgery.

**Methods:**

Step count data were collected from 244 patients who underwent minimally invasive surgery for lung cancer using Mi Band 5 to track preoperative and 3-day postoperative activity. Patients’ quality of life was assessed using the 12-Item Short Form Health Survey (SF-12) preoperatively and at 1 and 3 months postoperatively. Correlation and regression analyses were conducted to evaluate the impact of perioperative step count on hospital length of stay and quality of life.

**Results:**

Preoperative (*r* = −0.146, *p* = 0.023) and postoperative day 1 (*r* = −0.172, *p* = 0.018) step count were significantly correlated with the length of hospital stay. Postoperative day 1 step count was positively correlated with changes in SF-12 Physical Component Score (PCS) at 1 month (*r* = 0.186, *p* = 0.013). Pain significantly affected PCS changes at both 1 (*β* = −3.33, *p* < 0.001) and 3 months (*β* = −3.06, *p* < 0.001).

**Conclusion:**

Higher preoperative step counts are associated with a shorter hospital stay, while early postoperative physical activity is linked to both reduced hospital stay and improved short-term quality of life.

**Clinical trial registration:**

Clinicaltrials.gov, identifier NCT 04934657.

## 1 Introduction

The physical condition of patients affects surgical outcomes in multiple ways (1–4). While most previous influential studies have focused on patients’ chronic health problems (5–9), recent studies suggested that the physical activity is also linked to surgical outcomes (4, 10). Perioperative exercise has been shown to benefit surgical patients by improving their physical and functional status, partly by reducing systemic inflammation and improving cardiopulmonary reserve (11, 12). Walking is a central component of physical activity (13). Daily step count is widely accepted as metric for quantifying physical conditions (14–16). Although substantial evidence supports an association between step count and surgical outcomes (17–19), further exploration of key research questions is necessary.

The first question concerns the optimal timing for collecting step count data. Many studies have focused on postoperative step counts and their impact on length of hospital stay (LOHS) and recovery (19–21), while only a limited number have examined preoperative step counts (22). In this study, we collected step count data perioperatively to assess whether step counts at different phases (pre- and postoperative) influence surgical outcomes to varying degrees.

The second question relates to data analysis methods. Previous studies have often categorized patients into arbitrary step count groups (low, medium, and high) to compare clinical outcomes (18, 22). In contrast, we applied regression analyses to investigate the contribution of both preoperative and postoperative step counts to short-term and long-term surgical outcomes. This approach allowed for a more precise examination of how step count influences recovery at different stages.

The third question concerns case selection. Prior studies included heterogeneous patient populations undergoing various types of surgery (18, 23, 24), potentially limiting the ability to detect the impact of step count due to variability in patient and procedural factors. To address this limitation, our study focused exclusively on patients undergoing minimally invasive surgery for lung cancer. Lung cancer is one of the most prevalent cancers and a leading cause of cancer-related death worldwide, including in China (25). Pulmonary function assessment is crucial in the management of lung cancer patients, and walking directly affects pulmonary function, making lung cancer an ideal model for examining the relationship between step count and surgical outcomes.

Finally, step count data are often collected through patient self-reports, which are subject to recall bias. To enhance accuracy, we utilized the Mi Band, a wearable device, to objectively track step counts before and after lung segmentectomy procedures (14, 21, 26, 27).

In brief, this study aimed to quantify the relationship between perioperative step counts and surgical outcomes in patients undergoing minimally invasive lung cancer surgery. We anticipated that increasing preoperative step count would show a significant association with the improvement of short-term surgical outcomes, while increasing postoperative step count would be significantly associated with both short-term and long-term recovery.

## 2 Methods

This study was a sub-analysis of a prospective observational trial investigating the association between perioperative step count and recovery in patients undergoing elective major surgery (Clinical Trial Registration: NCT04934657). The study protocol was reviewed and approved by the local ethics committee (Approval No. 0569/2021). All participants provided written informed consent before enrollment, and patient identities were anonymized after data collection. This study has been reported in accordance with the STROCSS criteria (28).

### 2.1 Patients

We recruited patients (aged ≥18 years) who underwent minimally invasive surgery for lung cancer at our hospital between June 2021 and January 2022. Patients with an *American Society of Anesthesiologists (ASA)* physical status of I - III were included. Exclusion criteria included an inability to walk due to physical limitations or refusal to use the wearable device.

Upon providing informed consent, participants were required to wear a *Mi Band 5* (Xiaomi Technology Inc., Beijing, China) to track their step count before and after surgery. Patients with at least one valid preoperative step count were included in the analysis.

### 2.2 Perioperative care

All participants received standardized perioperative care following the *Enhanced Recovery After Surgery (ERAS)* program, which has been implemented at our institution since 2012. The ERAS protocol includes preoperative risk assessment by anesthesiologists and patient education by nurses. Anesthesiologists and surgeons collaborate to minimize the use of drains, reduce blood loss, and implement goal-directed fluid administration.

In the post-anesthesia care unit, early extubation of the endotracheal tube is prioritized. Pain management is standardized, with anesthesiologists performing thoracic paravertebral nerve blocks in combination with controlled intravenous analgesia. The acute pain service team ensures continuous and effective postoperative pain management. Additionally, postoperative respiratory therapy, early oral nutrition, and early mobilization are implemented to support recovery.

### 2.3 Step tracking

The *Mi Band 5* is equipped with a three-axis accelerometer that captures motion signals to calculate step counts during walking and jogging (29, 30). Patients were instructed to maintain their usual walking and exercise routines during the preoperative data collection period. Step counts were passively monitored throughout hospitalization. Activity monitors were recharged as needed, typically every 5–7 days. The duration of device usage per day was recorded for each patient, with a minimum requirement of 8 h per day for valid data entry.

Preoperative step counts were recorded for a minimum of three consecutive days before surgery. Although this may not fully reflect patients’ long-term habitual activity, it captures their physical status during the critical perioperative period and represents a feasible approach in real-world clinical settings.

### 2.4 Clinical data

Clinical data for each patient were collected during the preoperative consultation at the anesthesia clinic. Additional data were extracted from medical records, including age, sex, weight, and height. Health-related variables such as smoking status, alcohol consumption, and coexisting conditions (e.g., hypertension, diabetes, cardiovascular disease, chronic obstructive pulmonary disease) were recorded. The *Charlson Comorbidity Index (CCI)* was used to assess preoperative comorbidity levels, while the ASA physical status classification was used to evaluate preoperative physical status and surgical risk.

Surgery-related variables included tumor location and grade, preoperative spirometry results, and lung function data. Intraoperative data, such as the type of operation, extent of resection, operative time, and estimated blood loss, were collected to assess surgical performance. Short-term postoperative recovery variables included in-hospital complications, *length of hospital stay (LOHS)*, and total healthcare expenses related to surgery. Recorded postoperative complications included pneumonia, pleural effusion, atelectasis, infection, severe bleeding, cardiovascular events, embolic events, and critical organ dysfunction. The duration of chest drain catheter insertion was also documented as an indicator of postoperative recovery.

### 2.5 Outcomes

LOHS was retrieved from the hospital administration system and measured in hours, starting from the end of surgery until the discharge order was executed by the nurse. LOHS was reported in hours rather than days, as most lung cancer patients are discharged within one or two days after surgery, making day-based reporting insufficiently sensitive to capture meaningful differences between patients.

Postoperative complications were monitored for three months after discharge. Quality of life was assessed at three time points: preoperatively (T0), one month postoperatively (T1), and three months postoperatively (T2), using the *12-item Short-Form Health Survey (SF-12)*. The SF-12 is a validated instrument for evaluating both physical and mental health to assess patients’ overall quality of life (31). Pain intensity was evaluated at the same time points (T0, T1, T2) using the Numerical Rating Scale (NRS) to monitor postoperative pain trajectories and their potential influence on recovery outcomes.

### 2.6 Statistical analysis

Descriptive statistics, including means, medians, and proportions, were used to summarize demographic and clinical characteristics based on patients’ physical activity levels. Repeated measures *analysis of* var*iance* (ANOVA) was used to analyze longitudinal data. Scatter plots were generated to visualize correlations between step count and clinical outcomes. Pearson and Spearman correlation coefficients were calculated to quantify associations between step count and multiple clinical variables.

Regression analysis was performed to assess the impact of perioperative step count on LOHS and changes in quality of life (SF-12 scores). The primary variables of interest were the average preoperative daily step count and the step count on postoperative day 1. Variables with *p* < 0.05 or those deemed clinically relevant to surgical outcomes were included in the multivariate linear regression model. Each analysis included all available cases with complete data for the relevant variables; no imputation was performed.

All statistical analyses were conducted using *SPSS* software (Version 26.0, IBM Corporation, Armonk, NY, USA). A *p*-value < 0.05 was considered statistically significant. Results are reported as mean ± standard deviation (SD) or median and interquartile range (IQR), unless otherwise specified.

## 3 Results

### 3.1 Descriptive analysis

**Figure 1:**
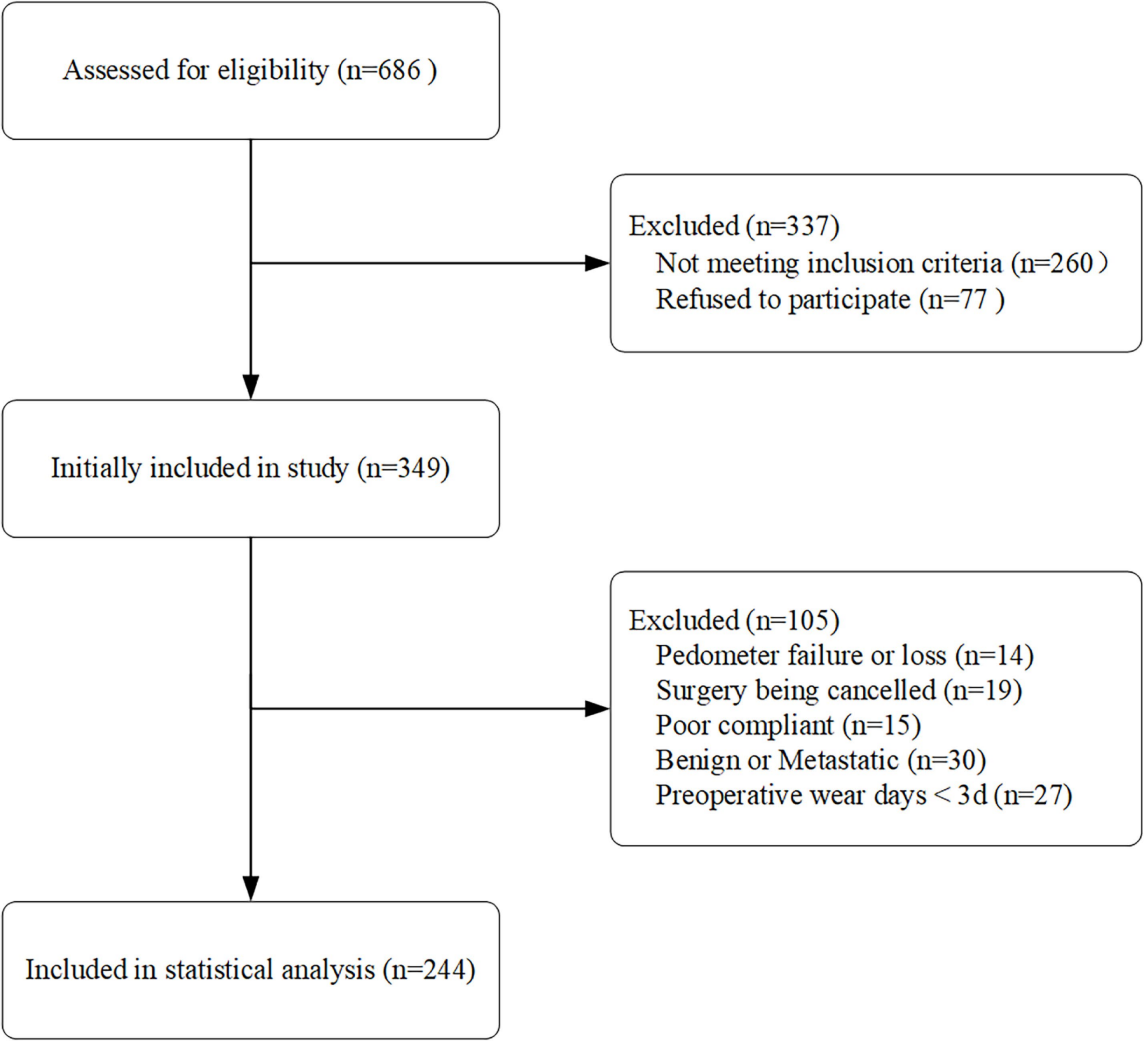
Flow diagram of patient recruitment.

We identified a total of 686 lung cancer patients. After screening, 260 patients were excluded, including 77 who declined participation, 19 whose surgeries were canceled, and 30 with benign or metastatic cancer. Additionally, 56 patients were excluded due to Mi Band 5 damage (*n* = 14), incomplete step count data (*n* = 27), or disqualification (*n* = 15). Ultimately, 244 patients were included in the statistical analysis (Figure 1).

Baseline demographic and surgical characteristics are presented in Table 1. The mean patient age was 56 ± 14 years, with women comprising 64.8% of the cohort. Most patients had preexisting chronic health conditions, with a median CCI of 2 (IQR: 2–3). ASA status I was recorded in 35.7% (87/244) of patients. The median preoperative SF-12 physical component score (PCS) was 55 (IQR: 53–56), while the median SF-12 mental component score (MCS) was 56 (IQR: 52–58).

**Table 1:** Patient demographics, preoperative conditions and surgical description.

Characteristic	Summary statistics (*N* = 244)
Preoperative characteristics	
Age, mean (SD), yr	56 (14)
Sex, No. (%)	
Female	158 (64.8)
Male	86 (35.2)
Body Mass Index, mean (SD), kg/m^2^	22.8 (3.4)
Current smokers, No. (%)	22 (9.0)
Alcohol consumption, No. (%)	48 (19.7)
ASA physical status, No. (%)	
I	87 (35.7)
II	157 (64.3)
Charlson comorbidity index, median (IQR)	2 (2–3)
Preoperative comorbidities, No. (%)	
Hypertension	78 (32)
Diabetes	15 (6.1)
Cardiovascular disease	116 (47.5)
Chronic obstructive pulmonary disease	6 (2.5)
Forced expiratory volume in the first second (predicted%), median (IQR)	95.5 (88.0–108.1)
Preoperative blood pressure, median (IQR), mmHg	
Systolic	126 (115–140)
Diastolic	75 (68–81)
Preoperative heart rate, median (IQR), beats/min	75 (70–83)
Preoperative SF-12 physical component score	55 (53–56)
Preoperative SF-12 mental component score	56 (52–58)
Preoperative pain NRS score, No. (%)	0 (0,1)
Location of tumor, No. (%)	
Left	92(37.7)
Right	152 (62.3)
Histopathology, No. (%)	
Adeno	222 (91)
Squamous	18 (7.4)
Others	4 (1.6)
TNM stage, No. (%)	
Tis	9 (3.7)
Ia	217 (88.9)
Ib	5 (2)
IIa	2 (0.8)
IIb	7 (2.9)
IIIa	4 (1.6)
Intraoperative characteristics	
Surgical approach, No. (%)	
Video-assisted thoracoscopic surgery	219 (89.8)
Robotic-assisted thoracoscopic surgery	25 (10.2)
Range of resection, No. (%)	
Sublobectomy	160 (65.6)
Lobectomy	84 (34.4)
Operation time, median (IQR), min	65 (45–90)
Intraoperative blood loss, median (IQR), ml	10 (5–20)
Postoperative characteristics	
Duration of chest drain catheter, median (IQR), hour	40 (24–46)
Length of hospital stay, median (IQR), hour	44 (37–49)
Hospitalization costs, median (IQR), ¥	36,254 (30015–44,059)
Postoperative complications, No. (%)	18 (7.4)
Pulmonary complication, No. (%)	10 (4.1)
Postoperative 1-month SF-12 physical component score^ψ^	50 (42,52)
Postoperative 1-month SF-12 mental component score*^ψ^*	57 (53,60)
Postoperative 3-month SF-12 physical component score^φ^	53 (45–56)
Postoperative 3-month SF-12 mental component score^φ^	56 (54–59)
Postoperative 1-month NRS^ω^	0 (0,1)
Postoperative 3-month NRS^φ^	0 (0,1)

Data are presented as mean ± SD, median (IQR) or number of patients (%). ASA, American society of anesthesiologists; NRS, numerical rating scale; TNM, tumor node metastasis.ψ:16 values missing; φ, 13 values missing; ω, 8 values missing.

All patients had non-small cell lung cancer, with adenocarcinoma being the predominant histology (91.0%, 222/244). The majority (91.0%, 222/244) were classified as TNM stage I. Sublobectomy (including wedge and segmental resections) was performed in 65.6% (160/244) of cases. All procedures were minimally invasive, with 89.8% (219/244) conducted via video-assisted thoracoscopic surgery (VATS) and 10.2% (25/244) via robotic-assisted thoracoscopic surgery (RATS).

The median operation time was 65 min (IQR: 45–90), with minimal intraoperative blood loss (median: 10 mL, IQR: 5–20). The median length of hospital stay (LOHS) was 44 h (IQR: 37–49), and the median duration of chest drain catheterization was 40 h (IQR: 24–46).

Postoperative complications occurred in 18 patients (7.4%), with pulmonary complications in 10 cases (4.1%), including pneumonia (*n* = 6), pleural effusion (*n* = 3), and atelectasis (*n* = 1).

### 3.2 Step count and SF-12

The median duration of device wear was five days preoperatively (IQR: 4–6) and two days postoperatively (IQR: 2–2). The median daily wear time was 22 h (IQR: 21–23) before surgery and 19 h (IQR: 18–23) postoperatively.

**Figure 2:**
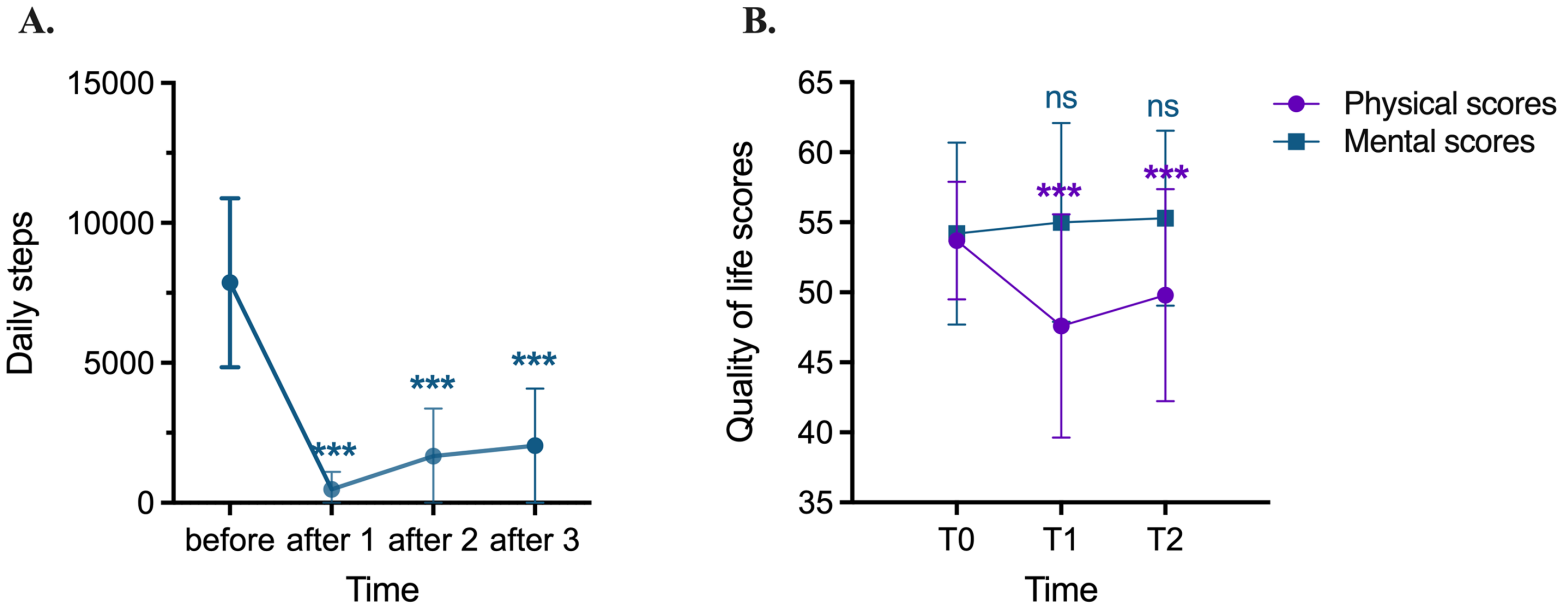
Daily steps and quality of life scores before and after surgery. **(A)** Daily steps before surgery and daily steps on days 1, 2 and 3 after surgery; **(B)** Quality of life scores before (T0), one month (T1) and three months (T2) after surgery, including physcial composition scores and mental composition scores. ***p < 0.001 (repeated measures-ANOVA).

All 244 patients completed preoperative step count monitoring, with a median daily step count of 7,233 (IQR: 5,399–9,247). Postoperatively, data availability varied due to inconsistent device use. On postoperative day 1, 191 patients recorded a median step count of 83 (IQR: 16–572). By postoperative day 2, 197 patients recorded 820 steps (IQR: 280–1,728), and by postoperative day 3, 186 patients recorded 1,151 steps (IQR: 415–2,509) (Table 2; Figure 2A).

**Table 2:** Patient step counts before and after the operation.

Step count	Pre-OP step *n* = 244	Pre-OP 5 *n* = 185	Pre-OP 4 *n* = 218	Pre-OP 3 *n* = 242	Pre-OP 2 *n* = 241	Pre-OP 1 *n* = 240	Post-OP 1 *n* = 191	Post-OP 2 *n* = 197	Post-OP 3 *n* = 186
Mean	7,585	7,362	7,464	7,579	7,945	8,216	404	1,327	1832
SDev	3,171	4,581	4,226	4,252	4,510	4,223	601	1,528	2017
Median	7,233	6,738	6,957	6,970	7,383	7,890	83	820	1,151
Minimum	935	301	94	893	772	424	0	0	0
Maxmum	21,023	26,188	24,240	26,883	23,320	30,556	2,857	7,568	9,832

Pre-OP, preoperative; Post-OP, postoperative; Pre-OP step count, sum of steps over three valid pre-operative days for each patient.

A total of 219 patients completed SF-12 assessments at both postoperative time points (Figure 2B). Compared to the preoperative baseline (median: 55, IQR: 53–56), the physical component score (PCS) significantly declined at one month postoperatively (median: 50, IQR: 42–52, *p* < 0.001). Although some recovery was observed at three months (median: 53, IQR: 45–56), PCS remained significantly lower than baseline (p < 0.001). The mental component score (MCS) did not show significant differences between preoperative and postoperative assessments (*p* > 0.05).

### 3.3 Correlation analysis

**Figure 3:**
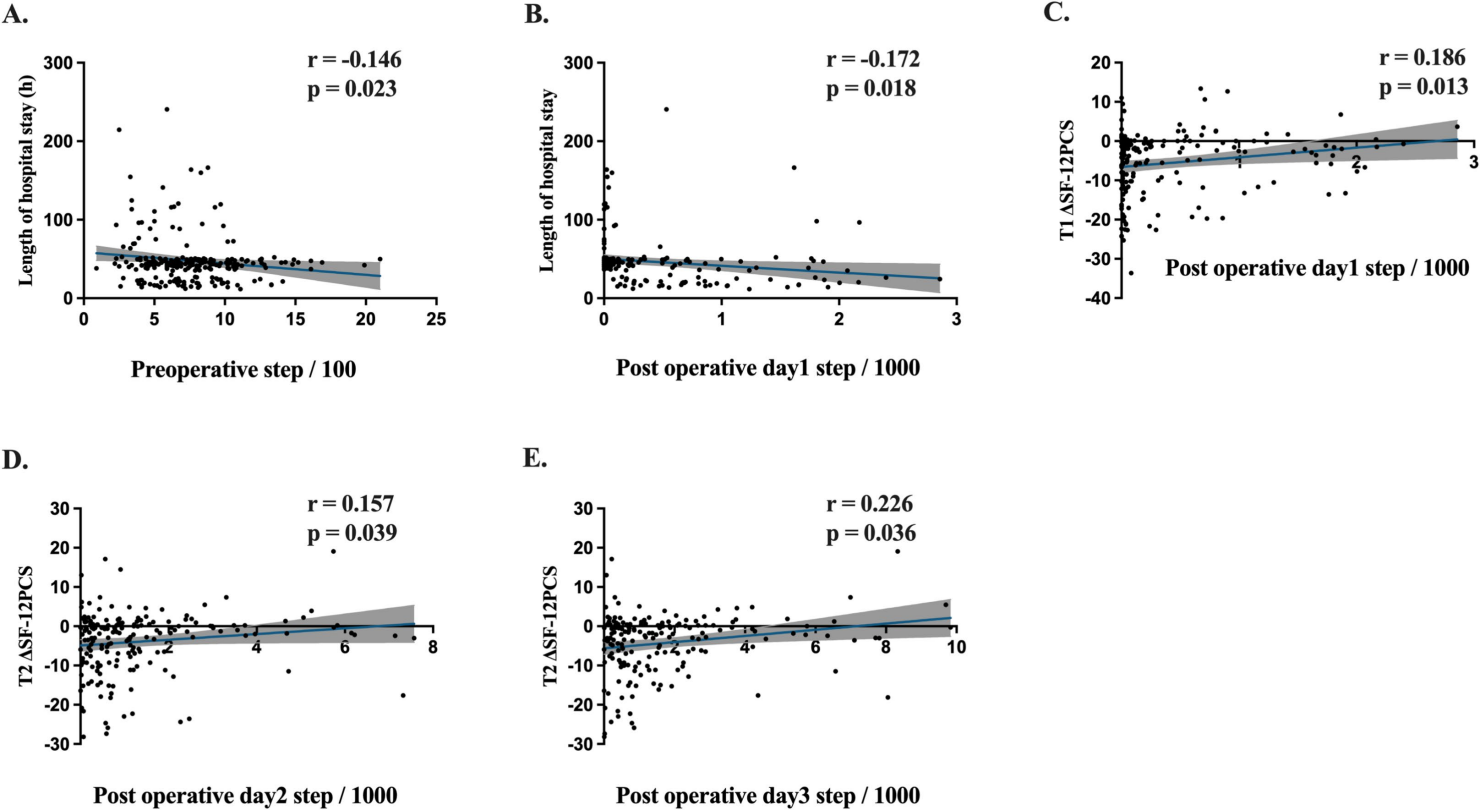
Pearson’s correlation coefficients (r), *p* values, regression line and 95% CI (gray shaded) are shown. **(A)** Preoperative step count and length of hospital stay correlation. **(B)** Post operative day 1 step count and length of hospital stay correlation. **(C)** Post operative day 1 step count and ΔSF-12PCS one month after surgery (T1) correlation. **(D)** Post operative day 2 steps and ΔSF-12PCS three month after surgery (T2) correlation. **(E)** Post operative day 3 steps and T2 ΔSF-12PCS correlation.

Scatter plots were constructed to examine the relationship between perioperative step count and postoperative outcomes, including LOHS and SF-12 PCS (Figure 3). Both preoperative step count and postoperative day 1 step count showed a weak but significant negative correlation with LOHS (*r* = −0.146, *p* = 0.023; *r* = −0.172, *p* = 0.018) (Figures 3A,3B).

To assess physical recovery, we analyzed the difference in SF-12 PCS (ΔSF-12 PCS = postoperative SF-12 PCS – preoperative SF-12 PCS) at one (T1) and three months(T2) postoperatively. A significant correlation was found between step count on postoperative day 1 and ΔSF-12 PCS at one month (T1, *r* = 0.186, *p* = 0.013) (Figure 3C). Additionally, step counts on postoperative days 2 and 3 significantly correlated with ΔSF-12 PCS at three months (Figures 3D,E). However, preoperative step count did not significantly correlate with ΔSF-12 PCS at either time point.

### 3.4 Regressions analysis

Table 3 presents the results of univariate and multivariate analyses for LOHS and ΔSF-12 PCS. Univariate analysis identified seven factors influencing LOHS: age, sex, Charlson Comorbidity Index (CCI), extent of resection, smoking status, preoperative step count, and postoperative day 1 step count. Multivariate regression indicated that sex, extent of resection, and postoperative day 1 step count were independent predictors of LOHS.

**Table 3:** Results of univariate and multivariate logistic regression analysis.

Outcome	Variables	Univariable analysis			Multivariable analysis		
		*β*	95%CI	*P*	*β*	95%CI	*P*
**LOHS**	Age	0.46	0.18~0.74	0.001	0.15	−0.17~0.47	0.360
	Male	19.99	12.17 ~ 27.80	<0.001	14.4	5.21 ~ 23.58	0.002
	BMI	−0.69	−1.86 ~ 0.47	0.245			
	CCI	7.90	1.03 ~ 14.77	0.025	2.57	−5.06 ~ 10.21	0.509
	Range of resection						
	Lobectomy	22.22	14.45 ~ 29.99	<0.001	17.56	8.78 ~ 26.34	<0.001
	Post-OP1 NRS	−0.62	−4.09 ~ 2.84	0.725			
	Smoking	18.02	4.51 ~ 31.53	0.01	8.44	−7.21 ~ 24.10	0.292
	Pre-OP step	−1.43	−2.66 ~ −0.21	0.023	−0.70	−1.92 ~ 0.51	0.257
	Post-OP1 step	−0.01	−0.02 ~ −0.01	0.018	−0.01	−0.01 ~ −0.01	0.024
**T1** **ΔSF-12PCS**	Age	−0.05	−0.13 ~ 0.02	0.187			
	Male	−0.04	−2.22 ~ 2.14	0.972			
	BMI	−0.23	−0.54 ~ 0.08	0.143			
	CCI	0.19	−1.65 ~ 2.03	0.839			
	Range of resection						
	Lobectomy	−0.74	−2.93 ~ 1.45	0.508			
	NRS (T1)	−3.70	−4.76 ~ −2.64	<0.001	−3.33	−4.64 ~ −2.01	<0.001
	Smoking	0.22	−3.41 ~ 3.86	0.904			
	Pre-OP step	−0.13	−0.46 ~ 0.20	0.436			
	Post-OP1 step	0.01	0.01 ~ 0.01	0.028	0.01	0.01 ~ 0.01	0.021
**T2** **ΔSF-12PCS**	Age	0.00	−0.07 ~ 0.07	0.937			
	Male	−0.58	−2.64 ~ 1.48	0.581			
	BMI	−0.29	−0.58 ~ −0.01	0.049	−0.33	−0.62 ~ −0.04	0.027
	CCI	0.48	−1.26 ~ 2.22	0.589			
	Range of resection						
	Lobectomy	−1.33	−3.39 ~ 0.74	0.209			
	NRS (T2)	−3.05	−4.24 ~ −1.86	<0.001	−3.06	−4.24 ~ −1.88	<0.001
	Smoking	−1.03	−4.47 ~ 2.41	0.557			
	Pre-OP step	−0.02	−0.33 ~ 0.30	0.922			
	Post-OP1 step	0.00	−0.00 ~ 0.00	0.144			

LOHS, length of hospital stay; ΔSF-12PCS, Postoperative SF-12 physical component score – Preoperative SF-12 physical component score; T1, one month postoperative; T2, three month postoperative; Post-OP1 NRS, postoperative day1 numerical rating scale; BMI, body mass index; CCI, the charlson comorbidity index; Smoking, current smokers; Pre-OP step, preoperative step count/1000; Post-OP1 step, postoperative day1 step count/1000. CI, confidence interval.

At one month postoperatively, pain was a significant predictor of ΔSF-12 PCS (*β* = −3.70, *p* < 0.001), while postoperative day 1 step count was positively associated with ΔSF-12 PCS (*β* = 0.33, *p* = 0.028). However, preoperative step count was not a significant predictor (*β* = −0.13, *p* = 0.436) (Table 3).

At three months, pain remained the dominant factor influencing ΔSF-12 PCS (*β* = −3.06, p < 0.001), while BMI also showed a significant association (*β* = −3.05, *p* = 0.027). Neither preoperative nor postoperative day 1 step count significantly predicted ΔSF-12 PCS at this time point. However, step count on postoperative days 2 (*β* = 0.88, *p* = 0.016) and 3 (*β* = 0.90, *p* = 0.001) were significant predictors of improved SF-12 PCS (Supplementary Appendix Table 1).

## 4 Discussion

The of our study support our hypothesis that step count before and after surgery has distinct impacts on surgical outcomes and patient recovery. Preoperative step count had a minimal effect on hospital length of stay (LOHS) and did not significantly influence changes in quality of life as assessed by the SF-12. In contrast, postoperative step count demonstrated a significant association with both LOHS and changes in the SF-12 Physical Component Score (ΔSF-12 PCS). These findings align with previous reports suggesting that increased physical activity, as measured by step count, benefits patients undergoing lung cancer surgery by reducing postoperative hospital stay and promoting better physical recovery (22).

We present new findings from this project. First, contrary to our expectations, preoperative step count was not strongly associated with postoperative outcomes. This may be attributed to the short tracking period, as we only recorded step counts for an average of three days before surgery. This timeframe may not adequately reflect a patient’s baseline physical activity level. Consequently, this limitation could have attenuated the observed associations between preoperative physical activity and postoperative recovery outcomes. Additionally, our study population primarily consisted of middle-aged patients (mean age: 56 years) undergoing minimally invasive surgery. Younger patients generally have better baseline health, higher physical fitness, and greater habitual physical activity, which could mitigate the influence of preoperative step count on postoperative recovery. Future studies should incorporate a more diverse patient population and different surgical procedures to provide a more comprehensive understanding of the role of preoperative physical activity in recovery.

Second, postoperative step count showed a significant association with key clinical outcomes, including LOHS and short-term quality of life. While our study does not establish causality and early mobility may reflect rather than promote recovery, existing evidence suggests that early postoperative mobility can reduce complications such as pulmonary infections and deep vein thrombosis (32). Encouraging patients to ambulate as early as possible after surgery may yield promising benefits for recovery.

Third, our findings suggest a synergistic relationship between preoperative and postoperative step counts, both contributing to certain surgical outcomes, such as LOHS. This reinforces the idea that physical activity should be emphasized both before and after surgery. Future randomized controlled trials are needed to evaluate the benefits of structured walking programs for surgical patients.

Several other clinical findings warrant discussion. Patients who underwent lobectomy had longer hospital stays than those who underwent sublobectomy. This underscores the importance of carefully determining the extent of lung resection to optimize patient outcomes. Additionally, we found that mid- to long-term postoperative pain significantly impacted physical recovery. Pain can reduce mobility, limit daily activities, and lead to muscle weakness and joint stiffness, ultimately hindering recovery (33). Implementing a comprehensive pain management strategy and providing holistic support could enhance patient recovery and improve long-term surgical outcomes.

Our study reported fewer postoperative complications and shorter hospital stays than previously published data (22, 34). We attribute this to the exclusive use of minimally invasive surgical techniques (VATS or RATS) and the setting of a high-volume tertiary hospital with a structured preoperative evaluation process. Enhanced preoperative preparation and perioperative care likely contributed to these positive outcomes.

Despite its strengths, this study has several limitations. First, although we adjusted for key demographic factors, comorbidities, and ASA physical status, the results may still be influenced by unmeasured residual confounders. Second, all patients in this study underwent minimally invasive surgery and were generally in good preoperative health, limiting the generalizability of our findings to patients with more advanced lung cancer or those undergoing major thoracic surgery. Third, the step count measured by the Mi Band 5 does not account for other important mobility metrics such as step length, walking patterns, or additional physical exertion factors like body load, which may affect the accuracy of physical activity assessment. Fourth, although participants were instructed to maintain their usual physical activity levels, there is a possibility that they altered their behavior before or after surgery, leading to discrepancies between recorded step counts and actual physical activity.

In addition, a *post hoc* power analysis was conducted using G*Power based on the observed effect sizes and sample size. The power values were 0.6 for the correlation between preoperative step count and LOHS, 0.7 for postoperative day 1 step count and LOHS, and 0.84 for postoperative day 1 step count and 1-month ΔSF-12 PCS. These moderate to adequate power levels support the reliability of our significant findings, although the somewhat limited power in some analyses suggests that future studies with larger sample sizes are needed to confirm and strengthen these associations.

In conclusion, preoperative and postoperative step counts have distinct effects on surgical outcomes. While preoperative step count was only associated with LOHS, early postoperative physical activity was linked to both a shorter hospital stay and better short-term quality of life. Encouraging patients to remain physically active throughout the perioperative period may enhance recovery. With the increasing adoption of wearable devices, future studies can leverage continuous step count data to provide a more detailed assessment of patients’ physical status. Integrating this data into clinical decision making such as risk stratification, personalized rehabilitation, and discharge planning has great potential to further optimize surgical outcomes and improve recovery. Based on our findings, randomized controlled trials should evaluate structured perioperative physical activity programs across diverse patient populations and surgical types, further exploring the relationships between physical activity, pain, and other recovery factors to provide stronger evidence for perioperative management.

## 5 Conclusion

Step counts before and after surgery had different effects on postoperative outcomes. The preoperative step count exhibited its influence solely on the LOHS. In contrast, the postoperative step count wielded a noteworthy dual influence, significantly affecting both the LOHS and the quality of life in the postoperative phase. These positive impacts on patient outcome highlights the importance of maintaining physical activity throughout the perioperative period.

## Data Availability

The raw data supporting the conclusions of this article will be made available by the authors, without undue reservation.
