# Supplementary material for: Association of perioperative step count tracked by a wristband with surgical outcomes in minimally invasive lung cancer surgery: a prospective observational study
Source: Front Med (Lausanne). 2025 Jul 29;12:1590327. doi: 10.3389/fmed.2025.1590327 (PMC12339247; doi:10.3389/fmed.2025.1590327)
Supplement: Supplementary file 1 [file Table_1.docx]

**Appendix tables**

aTable 1. Multivariate analysis of ΔSF-12 PCS at T2 including postoperative days 2 and 3 step count.

aTable 1. Multivariate analysis of ΔSF-12 PCS at T2 including postoperative days 2 and 3 step count.

|  | Variables | β | 95%CI | *p* |
| --- | --- | --- | --- | --- |
| Mode1 | BMI | -0.36 | -0.68 ~ -0.03 | 0.031 |
|  | NSR(T2) | 2.55 | -3.92 ~ -1.19 | <0.001 |
|  | Post-OP2 step | 0.88 | 0.17 ~ 1.59 | 0.016 |
| Mode2 | BMI | -0.34 | -0.66 ~ -0.03 | 0.031 |
|  | NSR(T2) | -2.83 | -4.20 ~ -1.47 | <0.001 |
|  | Post-OP3 step | 0.90 | 0.36 ~ 1.43 | 0.001 |

ΔSF-12PCS = Postoperative SF-12 physical component score – Preoperative SF-12 physical component score; T2: three month postoperative; NRS: numerical rating scale; BMI: body mass index; Post-OP step: postoperative day step count/1000. CI: confidence interval.
